# The complete mitochondrial genome of *Porodaedalea mongolica* (Hymenochaetaceae, Basidiomycota)

**DOI:** 10.1080/23802359.2022.2078677

**Published:** 2022-06-01

**Authors:** Heng Zhao, Xiao-Yong Liu, Fang Wu

**Affiliations:** a School of Ecology and Nature Conservation, Institute of Microbiology, Beijing Forestry University, Beijing, China; b College of Life Sciences, Shandong Normal University, Jinan, China; c State Key Laboratory of Mycology, Institute of Microbiology, Chinese Academy of Sciences, Beijing, China

**Keywords:** Polypores, Hymenochaetales, mitogenomic data, phylogeny, wood-inhabiting fungi

## Abstract

Most *Porodaedalea* species are important phytopathogenic and medicinal fungi. Recently, several *Porodaedalea* species including *P. mongolica* were newly described. In the present study, the complete sequence of mitochondrial genome of *P. mongolica* was determined, with a size of 114,176 bp and a GC content of 28.98%, containing two ribosomal RNA subunit, 26 transfer RNA, and 54 protein-coding genes (PCGs). The comparative analyses indicated that the amino acids of 14 core PCGs were highly conserved in *Porodaedalea*. Phylogenetic analysis of *Porodaedalea* was performed based on mitogenomic data and provided a new insight to the phylogeny of the *Porodaedalea*. The complete mitogenome sequence provides important data for further study of *Porodaedalea.*

The polypore genus *Porodaedalea* belongs to Hymenochaetales in Basidiomycota, and widely distributes in Europe, North America, North Africa, and Asia (Dai 2010; Wu et al. 2019a). Some species in this genus attack the heartwood of living conifers and cause a white rot, while others are important medicinal resources (Wu et al. 2019a, 2019b). *Porodaedalea mongolica* Y.D. Wu and Y. Yuan was found on living trees of *Larix gmelinii* in China (Wu and Yuan 2020). A living strain Dai 20809 was cultivated from its fruiting bodies and preserved at the herbarium of Institute of Microbiology, Beijing Forestry University (BJFC). Mycelia were harvested after an incubation at 27 °C for two weeks on PDA plates (20 g/L agar, 20 g/L glucose, 200 g/L potato, and 1000 mL distilled water), and cell DNA was extracted using the CTAB method (Watanabe et al. 2010). The whole genomic sequencing was performed by Illumina HiSeq 4000 platform (Illumina Inc., San Diego, CA) following the manufacturers’ instructions with Novogene (https://novogene.com/, Beijing, China). Raw reads were quality-controlled to acquire clean data which in turn was used to assemble the mitogenome by NOVOPlasty 4.2.1 (Dierckxsens et al. 2017). The mitogenome was annotated automatically using Mfannot (http://megasun.bch.umontreal.ca/cgi-bin/mfannot/mfannotInterface.pl) and GeSeq (Tillich et al. 2017), and then manually annotated. The obtained clean data and assembled complete mitochondrial genome were deposited at GenBank under accession numbers SRR15959588 and OK217283, respectively.

The total length of mitogenome of *Porodaedalea mongolica* was 114,176 bp with a GC content of 28.98%, smaller than *P. pini* (144,970 bp with a GC 28.26, two rRNA genes, 26 tRNA genes, and 100 open read frames (ORFs), Lee et al. 2019). The mitogenome contains two rRNA genes (*rns* and *rnl*), 26 tRNA genes, and 54 protein-coding genes (PCGs) including 14 core ones of the electron transport and oxidative phosphorylation system, 49 free-standing ORFs and one DNA polymerase gene. Sixteen introns were annotated, two in *cob*, eight in *cox1*, one in *nad5* and five in *rnl*.

Amino acid sequences of the 14 core PCGs were employed to reconstruct phylogeny of polypore using the software RAxML (version 8) with 100 bootstrap replicates (Stamatakis 2014). The maximum-likelihood tree (Figure 1) follows previous studies (Nie et al. 2019, 2021), and supplement mitogenomic data of *Porodaedalea mongolica*. In the phylogram, eight species of six genera in Hymenochaetaceae are clustered into one clade (bootstrap = 100%). *Porodaedalea mongolica* is most closely related to *P. pini* (bootstrap = 100%), which demonstrates that 14 core PCGs are highly conserved in the genus *Porodaedalea*. Nevertheless, the mitochondrial genome also has variability in *Porodaedalea*, especially a remarkable increase in the number of introns (16 in *P. mongolica* versus 21 in *P. pini*, Lee et al. 2019), resulting in an expansion of mitochondrial genome (114,176 bp in *P. mongolica* versus 144,970 bp in *P. pini*, Lee et al. 2019). In addition, the ribosomal protein S3 gene (*rps3*) in *P. pini* was lost in *P. mongolica* (Lee et al. 2019).

**Figure 1.**
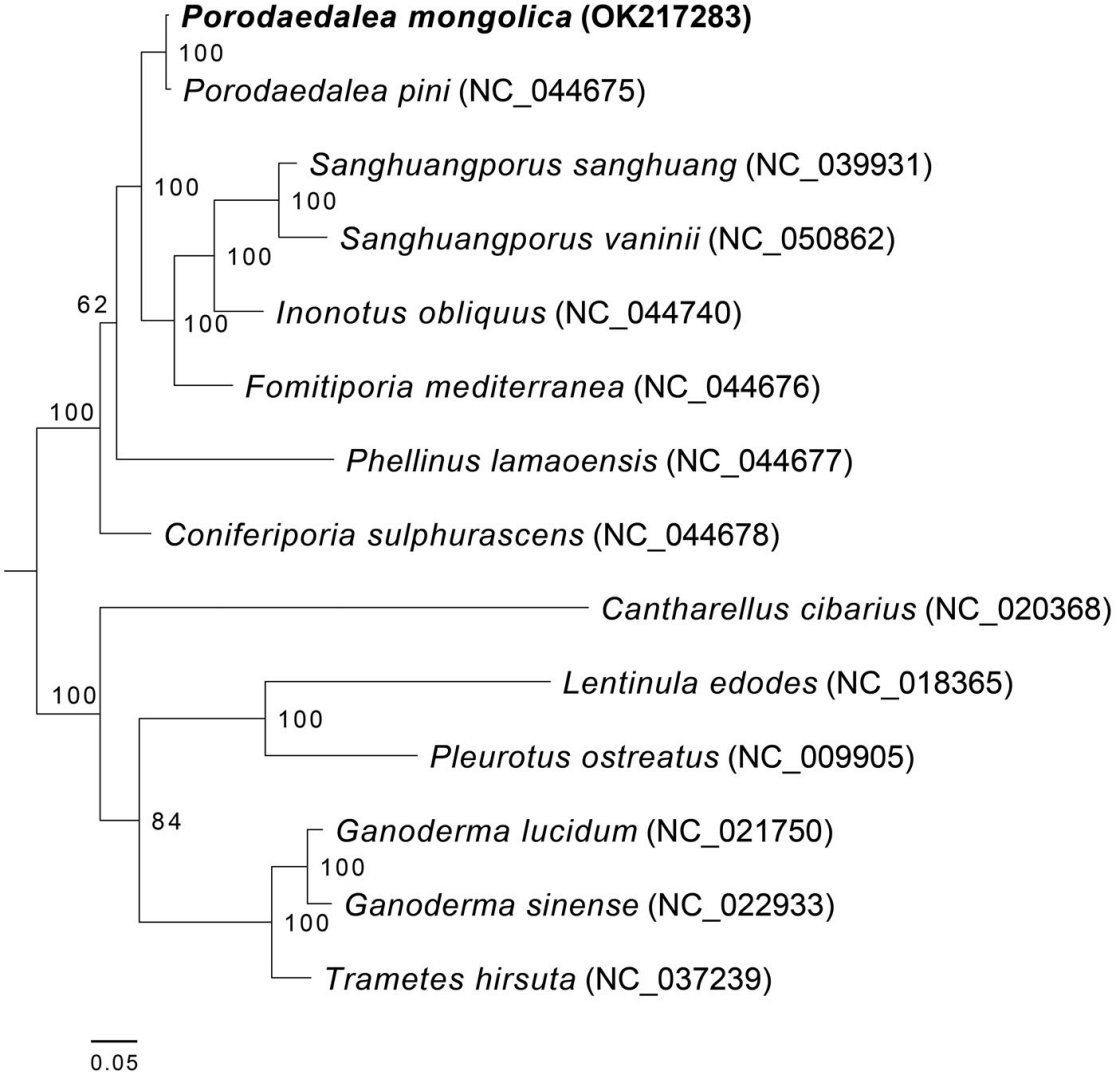
The maximum-likelihood phylogenetic tree based on amino acid sequences of 14 core mitochondrial proteins. These proteins contain ATP synthase subunits (*atp6*, *atp8*, and *atp9*), the apocytochrome b (*cob*), cytochrome oxidase subunits (*cox1*, *cox2*, and *cox3*), and NADH dehydrogenase subunits (*nad1*, *nad2*, *nad3*, *nad4*, *nad4L*, *nad5*, and *nad6*). Maximum-likelihood bootstrap values (≥50%) are indicated along branches. Mitogenomes were used in this phylogenetic analysis: *Cantharellus cibarius* (NC_020368), *Coniferiporia sulphurascens* (NC_044678), *Fomitiporia mediterranea* (NC_044676), *Ganoderma lucidum* (NC_021750), *Ganoderma sinense* (NC_022933), *Inonotus obliquus* (NC_044740), *Lentinula edodes* (NC_018365), *Phellinus lamaoensis* (NC_044677), *Pleurotus ostreatus* (NC_009905), *Porodaedalea mongolica* (OK217283, this study), *Porodaedalea pini* (NC_044675), *Sanghuangporus sanghuang* (NC_039931), *Sanghuangporus vaninii* (NC_050862), and *Trametes hirsute* (NC_037239). The bottom bar represents 0.05 changes per site.

## Data Availability

The data that support the finding of this paper are available in GenBank of NCBI at http://www.ncbi.nlm.nih.gov/, bio-project, bio-sample, SRA, and mitochondrial genome numbers PRJNA764329, SAMN21499892, SRR15959588, and OK217283, respectively.
